# Listening to the environment: hearing differences from an epigenetic effect in solitarious and gregarious locusts

**DOI:** 10.1098/rspb.2014.1693

**Published:** 2014-11-22

**Authors:** Shira D. Gordon, Joseph C. Jackson, Stephen M. Rogers, James F. C. Windmill

**Affiliations:** 1 Department of Electronic and Electrical Engineering, University of Strathclyde, Glasgow, UK; 2 Department of Zoology, University of Cambridge, Cambridge, UK

**Keywords:** epigenetic, hearing, insect, locust phase, neurophysiology, membrane mechanics

## Abstract

Locusts display a striking form of phenotypic plasticity, developing into either a lone-living solitarious phase or a swarming gregarious phase depending on population density. The two phases differ extensively in appearance, behaviour and physiology. We found that solitarious and gregarious locusts have clear differences in their hearing, both in their tympanal and neuronal responses. We identified significant differences in the shape of the tympana that may be responsible for the variations in hearing between locust phases. We measured the nanometre mechanical responses of the ear's tympanal membrane to sound, finding that solitarious animals exhibit greater displacement. Finally, neural experiments signified that solitarious locusts have a relatively stronger response to high frequencies. The enhanced response to high-frequency sounds in the nocturnally flying solitarious locusts suggests greater investment in detecting the ultrasonic echolocation calls of bats, to which they are more vulnerable than diurnally active gregarious locusts. This study highlights the importance of epigenetic effects set forth during development and begins to identify how animals are equipped to match their immediate environmental needs.

## 1. Introduction

How do animals adapt to a changing environment? Many animals show some degree of phenotypic plasticity, expressing alternative morphologies, physiologies or behaviours in order to be able to cope better with the environmental conditions they face [1,2]. This plasticity may occur as short-term alterations, induced by immediate conditions that may principally affect neuronal function or behaviour. Alternatively, more profound epigenetic changes may arise from an altered developmental trajectory, based on past environmental or even parental circumstances [3,4].

**Figure 1.**
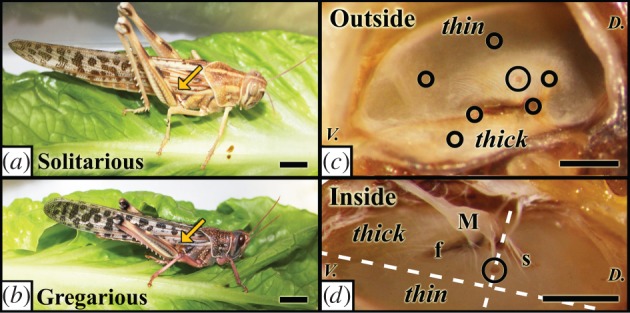
Adult (*a*) solitarious and (*b*) gregarious locusts with an (*c*) outside and (*d*) inside view of a tympanal membrane. The pyriform vesicle (PV) is in a larger circle for both (*c*) and (*d*), and the other circles represent the points on the membrane that were measured for displacement. In the inside view, Müller's organ (M) is intact to show the location of the neurons; other attachment points are the folded body (*f*) and the styliform body (s) and orientation is given as dorsal (*D.*) and ventral (*V.*). The direction of the travelling wave goes from the *thin* portion (proximal) of the membrane to the *thick* region (distal), peaking at the PV. Scale bar is 1 cm in (*a*,*b*) and 0.5 cm in (*c,d*). (Online version in colour.)

The desert locust *Schistocerca gregaria* shows an extreme phenotypic plasticity, which exhibits a trans-generational accumulation of phenotypic change that is driven by changes in population density and so is a known example of different morphologies due to epigenetics [5–7]. The two extreme phenotypes are called the solitarious and gregarious phases (figure 1*a,b*) and they differ extensively in behaviour, physiology and morphology [8]. At low population densities, locusts tend to exist in the solitarious phase. They are cryptic in coloration and behaviour, moving infrequently and with a characteristic creeping gait [9]. Under most circumstances, they actively avoid other locusts, dispersing themselves widely in the environment. When they undertake long-distance flights, they do so under the cover of darkness at night [10,11]. An increasing population density, which leads to forced contact with other locusts, triggers the transformation to the gregarious phase. Gregarious locusts are highly active, conspicuous in both behaviour and appearance with aposematic coloration as larvae [12]. Most importantly, they actively aggregate into large migratory swarms that may ultimately consist of billions of individuals. Phase change in the locust is a process that occurs over many different timescales: some critical behavioural changes occur within just a few hours [13,14], changes in coloration occur over a locust's lifetime, but full morphological change requires multiple generations [5].

The sensory systems differ between the two phases with solitarious locusts having larger eyes [15] and longer antennae with a greater number of olfactory sensilla than gregarious locusts [16]. Gregarious locusts, however, have a higher density of mechanoreceptors and contact-chemoreceptors on their legs [17]. It therefore appears that sensory structures involved in detection from a distance (sight and smell) are more heavily invested in by solitarious locusts, whereas gregarious locusts have greater numbers of receptors for proximate stimuli (touch and taste). Gregarious locusts live constantly amidst a throng of other locusts, which presents considerable sensory complexity [18] but possibly interferes with the detection of distant stimuli.

In common with other Acrididae, locusts have a well-developed auditory system that is able to determine a wide range of pitch [19–21]. Unlike other grasshoppers, however, desert locusts do not sing or apparently detect mates through sound. Locusts will take evasive action when flying in response to hearing the ultrasonic calls of hunting bats [22], and there are reports that the flight sound of fellow swarm members is a strong stimulus for locusts on the ground to take wing [23]—there is, however, no one clear reason for audition in locusts.

Nevertheless, locusts have a complex hearing organ. Locusts have two ears, located laterally on the first segment of the abdomen, often covered by the wings when resting, with the tympanal membrane on the outer surface (arrows, figure 1*a,b*). The tympanal membrane constitutes the principal sound receiver; it is approximately 1.5 × 2.5 mm and partially encased in a hard, sclerotized semicircle of cuticle embedding and protecting the membrane (figure 1*c*). The internal surface of the membrane is backed by an air sac, which enables the ear to act as a pressure difference receiver at low frequencies. The auditory nerve is enveloped by this air sac (figure 1*d*). There are approximately 70 afferent auditory neurons forming the auditory nerve, which contains no other type of neuron, and enters the central nervous system in the metathoracic ganglion [24]. Some of the auditory sensory neurons attach directly to the tympanic membrane at the pyrifom vesicle (PV), folded body and styliform body, while others attach indirectly via a structure called Müller's organ (figure 1*d*) [25]. Development of the hearing structures progresses with each instar, with the PV not forming until the third instar and the folded body during the fourth instar, despite the neurons being present from the first instar [26]. Detection and initial frequency determination occurs when sound causes the tympanum to vibrate and produce a travelling wave, initiating in the thin portion of the membrane and moving maximally at the PV [27]. This creates different levels of deflection across the membrane, resulting in distinct displacement and phase patterns in the neural attachment sites [28]. After peaking at the PV, the travelling wave becomes heavily attenuated, especially at high frequencies, in the thick part of the membrane. For examples of movies of travelling waves at three frequencies used throughout this manuscript (3, 10 and 15 kHz), see the electronic supplementary material, videos S1–S3.

We investigated whether there were phase-related differences in both locust-hearing mechanics and neural response. We hypothesized that the hearing of solitarious locusts may need to be more sensitive to detect predators from further away. We measured the morphology of the tympanum in both phases and determined its response to a broad spectrum of sound frequencies and intensities. We also used a comparative measure of overall neural activity in the auditory nerve to analyse auditory thresholds and frequency tuning in each phase.

## 2. Material and methods

### (a) Animals

Desert locusts, *S. gregaria* (Forskål), were reared to produce either gregarious or solitarious phenotypes using the husbandry techniques developed by Roessingh *et al*. [29], at the Department of Zoology, University of Cambridge. Gregarious locusts were maintained at a high population density (approx. 3000 m^3^). The solitarious locusts used in the experiments had been isolated from the main population for two or three generations and had been reared in individual cages that prevented visual or olfactory stimulation from other locusts. Animals were fed seedling wheat and wheat bran, and shipped overnight as newly moulted adults to the University of Strathclyde for experiments. The locusts were used in the experiments from 5 to 14 days post adult moult.

### (b) Morphology

The tympanal membrane was dissected from each animal after the laser vibrometry experiments. Photos were taken of each membrane through a dissecting microscope (Leica M80, Wetzlar, Germany) with a digital camera (Canon, EOS 550, Tokyo, Japan) and calibrated with a micrometre slide (Fine Science Tools, 29025-02, Heidelberg, Germany). The membranes were positioned as flat as possible, as any inclined angle would result in a skewed image for dimension analysis. Three photos were taken with different rotations to account for photo clarity and any slight offsets. Photos were then analysed using SolidWorks (Dassault Systemes, Waltham, MA, USA). Length was determined by measuring from the most posterior sclerotized portion to the furthest point on the curve of the tympanal membrane rim opposite (figure 1*d*). Width was then determined from creating a line perpendicular to the length vector through the PV. Finally, the area of the PV was also measured. Measurements are mean ± s.e.m.

### (c) Membrane deflections

The right wings were removed from the locusts, and the animals were restrained with Blu-tack (Bostik-Findley, Stafford, UK) in a natural position with their tympanal membrane exposed to a micro-scanning Laser Doppler Vibrometer (PSV 300, Polytec, Waldbronn, Germany) with a close up unit (OFV 056). A loudspeaker (Heil Air Motion Transformer, ESS, South El Monte, USA) was placed facing the ear to play sound to the locust, at least 10 cm away. A microphone (Bruel & Kjaer 4138, Naerum, Denmark) was positioned in close proximity to the locust's tympanum to measure the sound pressure at the tympanal membrane. A broadband linear chirp from 1 to 20 kHz was played at 65 dB sound pressure level (SPL; re 20 µPa at 10 cm) to the animals, generated by the laser vibrometer's control computer and then passed through an amplifier (TA-FE370, Sony, Tokyo, Japan). A scan was performed across the whole membrane to visualize the travelling wave (electronic supplementary material, videos S1–S3), in addition to measuring the deflection at specific locations of interest (figure 1*c*). The fast Fourier transform resolution was 12.5 Hz, and measurements averaged at least 15 times per point measured, with coherence above 85%, and later binned to 500 Hz categories. Gain (displacement/SPL) values were used for analysis to account for any differences in sound signal amplitude. Non-normally distributed data were log transformed before statistical analysis. The sample size was 19 gregarious and 11 solitarious locusts (15 males and 15 females).

### (d) Electrophysiology

#### (i) Preparation

All four wings were removed and the locust was mounted ventral-side up in dental beading wax (Kedment, DWS307, Purton, UK). More wax was used to immobilize the body and legs, leaving the meso- and metathoracic segments free as well as the first segment of the abdomen, so sound could reach the tympana unimpeded. A small window was cut through the sternum of the metathorax, and the ventral air sacs were removed. A pair of hook electrodes made from 50 μm silver wire was placed under the auditory nerve and insulated using petroleum jelly. Ringer's solution (NaCl 190 mM, KCl 2 mM, MgCl_2_ 4 mM, CaCl_2_ 4 mM, NaHPO_4_ 1 mM; pH 7.8) was used to keep the preparation moist, as needed [30]. The final sample size was 14 gregarious and 15 solitarious locusts (17 males and 12 females).

#### (ii) Sound stimulus

The preparation was located within a custom-built sound isolation box. Sound was played from a speaker (ESS Air Motion Transformer) placed facing the tympanal membrane from a distance of at least 10 cm. A reference microphone (Bruel & Kjaer 4138) was located near the tympanal membrane to determine SPL at the ear. The sound stimulus was created with a custom LabView (National Instruments, version 8.5.1; Austin, USA) program, fed through a data acquisition system (National Instruments USB-6251 and BNC-2110) and amplified (Sony TA-FE370). To ensure that all animals were at the same level of sensory adaptation, white noise (1–20 kHz) was played for 30 s before playing any of the experimental stimuli. A sequence of tapered cosine-windowed (Tukey-windowed) pure-tone bursts were then played, ranging from 1 to 20 kHz at 1 kHz intervals over a 60 dB (SPL) range from 40 to 100 dB with 1 dB step sizes. Each tone lasted 70 ms, followed by a 70 ms interstimulus interval of no sound. The sequence order was randomized and different for each experimental run. This was then repeated for a sequence of 1.25, 2.25 kHz and every 1–20.25 kHz, then again at 1.5, 2.5–20.5 kHz and finally at 1.75, 2.75–20.75 kHz. Each sequence was randomized again and repeated multiple times per animal.

#### (iii) Data analysis

**Figure 2.**
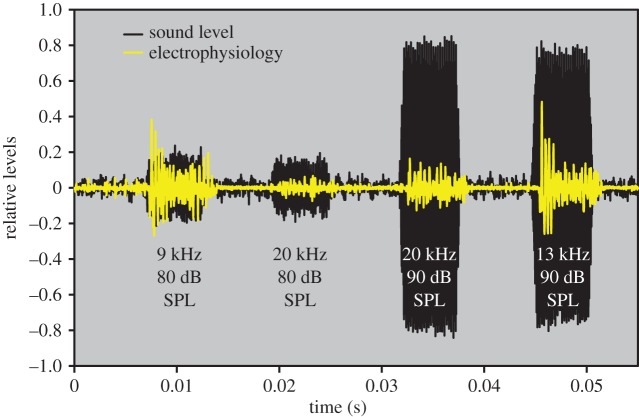
Sample overall electrophysiological response, where black is the relative microphone sound level, and shaded yellow is the relative electrophysiology response for frequencies and sound levels: 9 kHz (80 dB SPL), 20 kHz (80 dB SPL), 20 kHz (90 dB SPL) and 13 kHz (90 dB SPL). (Online version in colour.)

Data was processed in LabView. The neural response is the summed discharge of many individual sensory neurons in the auditory nerve; as such, this compound response cannot be broken down into the trains of spikes produced by single neurons in response to the sound stimuli (figure 2). What appear to be single action potentials in the recordings are compound events generated by the coherent firing of several neurons simultaneously. A measure of the overall neural responses was therefore produced by calculating the root mean square (RMS) of the signal for the duration of the sound stimulus. This gives an overview of the extent of spiking activity in the nerve. Figure 2 shows an example of four sound stimuli (black) of different sound levels and frequencies that result in different amplitudes of the electrophysiological response (shaded yellow). We also measured the latency to the onset of neural activity from the start of the stimulus. Latency was calculated to the time the signal first exceeded 20% of its maximum amplitude (each response was normalized to its maximum and then smoothed using a 100-point Savistky-Golay filter). Averaging across animals and repeated measurements mitigated type I and II errors typical of a thresholding system.

Each individual dataset (several within an animal), comprising the responses to a full frequency range of 1–20 kHz sound stimuli across all sound intensities, was normalized to the maximum amplitude of the RMS electrophysiological response in the dataset, to control for any change in signal intensity over time (excluding latency data, which remained as absolute values). The result of one experiment therefore comprised sound frequency and intensity as independent variables and the electrophysiological RMS as the dependent variables. All experimental files from one animal were pooled together and analysed to generate an auditory response map (electrophysiological response per frequency/decibel). Statistical tests were carried out in SPSS (IBM, Armonk, USA) using a one-way ANOVA to compare the electrophysiological response at each sound level per frequency. All data were reanalysed with sex as a grouping factor to identify any difference owing to sex and as a control for phase. To ensure no bias during the normalization process, we measured the maximal response level by comparing the RMS of the non-normalized maximal response with the signal with no sound—spontaneous electrical activity. Data are presented as mean ± s.e.m.

## 3. Results

### (a) Membrane morphology

The tympanum is near elliptical in shape (figure 1*c*), with the plane of the membrane approximately perpendicular to the anterior–posterior axis of the body, the long axis in line with the dorsal–ventral body axis, and the thin membrane more proximal to the midline. The short axis, width, of the tympanal membrane is 7.5% larger in solitarious locusts (solitarious: 1.56 ± 0.04 mm; gregarious: 1.45 ± 0.02 mm; *F*_1,28_ = 7.27, *p* = 0.012). The long axis is not significantly different between phases (solitarious: 2.84 ± 0.10 mm, gregarious: 2.69 ± 0.03; *F*_1,28_ = 2.762, *p* = 0.108). The width measurement corresponds to the approximate propagation direction of the travelling wave (electronic supplementary material, videos S1–S3). There is no difference in the area of the PV (solitarious: 0.13 ± 0.02 mm^2^, gregarious: 0.12 ± 0.01 mm^2^, *F*_1,28_ = 0.21, *p* = 0.65). When evaluating the data by sex, females have significantly longer tympanal membranes (female: 2.91 ± 0.06 mm, male: 2.58 ± 0.03 mm, *F*_1,28_ = 26.6, *p* < 0.001), but show no significant difference in their width (female: 1.52 ± 0.03 mm, male: 1.45 ± 0.03 mm, *F*_1,28_ = 2.98, *p* = 0.095). The PVs are not significantly different in area (female: 0.13 ± 0.01 mm^2^, male: 0.12 ± 0.01 mm^2^, *F*_1,28_ = 0.85, *p* = 0.37).

### (b) Membrane deflections

**Figure 3.**
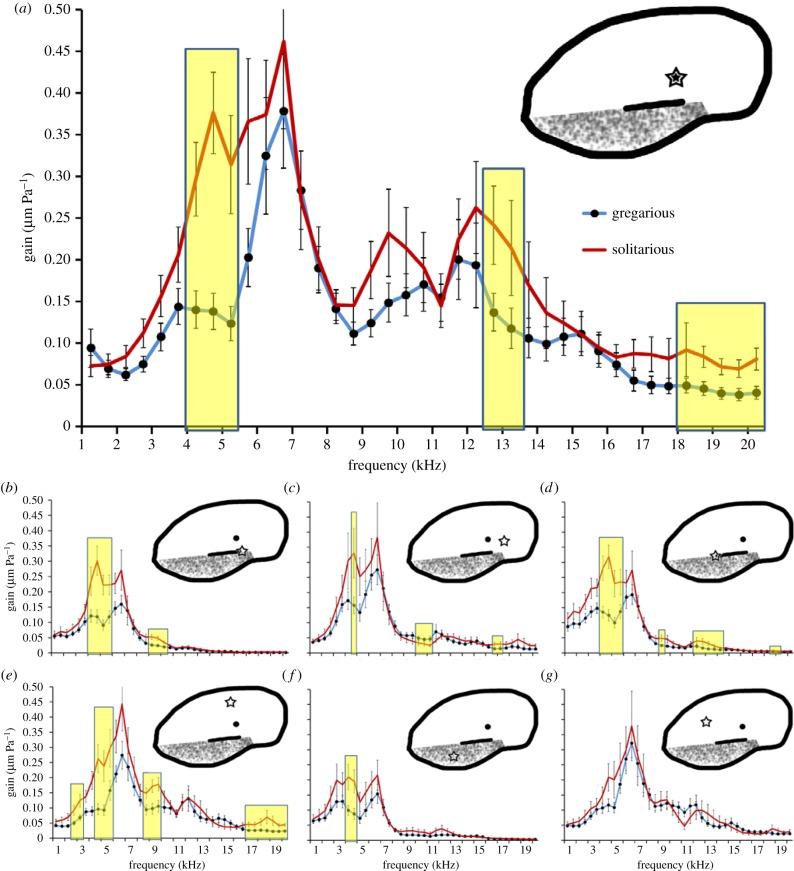
Tympanal movement as gain µm Pa^−1^ (to account for exact displacement with sound level) at different points on the membrane surface (indicated by the stars). The pyriform vesicle is shown at (*a*), with other main points of neuron attachment at (*b,c*). The bottom edge of the folded body is located at (*d*) and the origin of the travelling wave is at (*e*). The thick membrane is located at (*f*) and (*g*) is a point on the membrane not involved in the travelling wave or neuron attachment. Shaded yellow regions indicate significance of *p* < 0.05. (Online version in colour.)

Independent of phase, the tympanal membrane moves maximally at the PV (figure 3*a*). For all points of neural attachment (figure 3*a–d*) and along the travelling wave (figure 3*e,f*), there is a peak in the mechanical-response spectrum around 4–7 kHz, with the membrane moving significantly more in the solitarious locusts (figure 3 significance of *p* < 0.05 shaded in yellow). At any given frequency, a doubling of movement indicates a 6 dB difference in response. Therefore, for example, at 5 kHz the PV membrane has over double the displacement in solitarious locusts (solitarious 0.31 ± 0.06 µm Pa^−1^, gregarious 0.12 ± 0.02 µm Pa^−1^; *F*_1,28_ = 12.59, *p* = 0.001) indicating that the tympana of gregarious locusts would require a 6 dB louder signal to move the same amount as those of solitarious locusts.

For frequencies greater than approximately 10 kHz, there is reduced tympanal movement, for the points of neural attachment other than the PV (figure 3), as expected based on previous studies that measured the mechanical vibrations of the locust tympanum [27,28]. The PV of solitarious locusts displays greater membrane displacement than those of gregarious locusts for frequencies above 10 kHz (figure 3*a*). The lateral point chosen on the tympanum that does not fall in the path of the travelling wave or have any neural attachments underlying it (figures 1*c* and 3*g*) shows no difference between phases. No significant differences were found between the sexes at any of the locations or frequencies (e.g. 5 kHz at the PV: male 0.23 ± 0.05 µm Pa^−1^, female 0.16 ± 0.03 µm Pa^−1^; *F*_1,28_ = 0.26, *p* = 0.61).

### (c) Neural response

**Figure 4.**
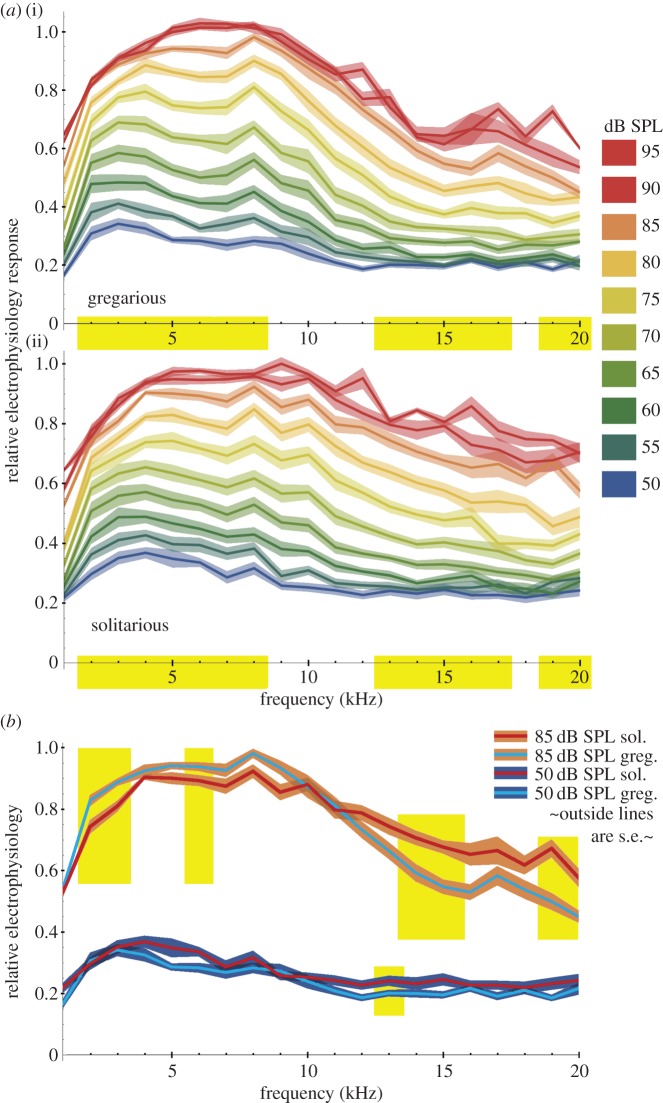
Electrophysiological response in the auditory nerve at different sound levels across a sound frequency range of 1–20 kHz. (*a*)(i) Gregarious locust (50–95 dB SPL) and (ii) solitarious locust (50–95 dB SPL). Shaded yellow indicates regions where at least some sound levels had significant differences (*p* < 0.05) between phases. (*b*) Gregarious and solitarious locusts at 50 and 85 dB SPL. Data are means with the outer shading indicating the standard error. Shaded yellow regions indicate significant differences at those specific frequencies for those sound levels (*p* < 0.05). (Online version in colour.)

**Figure 5.**
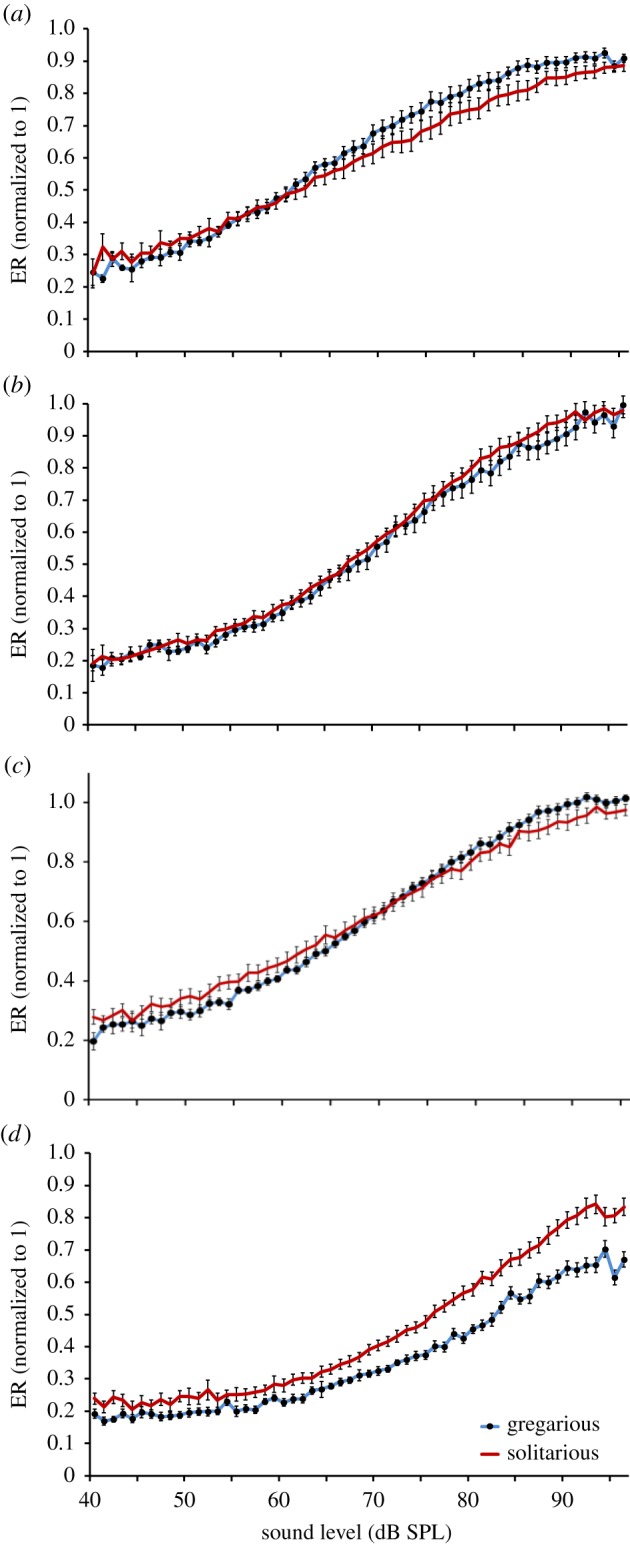
Electrophysiological response (ER) at four selected frequencies (*a*) 3, (*b*) 5, (*c*) 10 and (*d*) 15 kHz. Electrophysiology responses for each frequency do not always saturate at 1 owing to differential frequency sensitivity of the neuronal ensemble. The blue line with black dots is gregarious, and the red line is solitarious locusts. Data are means ± s.e. (Online version in colour.)

Louder sounds increased the neurophysiological response measured in the auditory nerve across all frequencies in both phases (figures 4 and 5). Both gregarious and solitarious locusts showed a similar response in that the peak response lay between frequencies 4–8 kHz (figure 4*a,b*). Data from each animal were normalized to its own maximal RMS response (across all sound levels and frequencies). The RMS of the maximum neural responses was 11.65 ± 1.15 times greater than the RMS of the same duration of spontaneous electrical activity when no auditory stimulus was being played. The maximum response relative to baseline activity before normalization was similar in both gregarious (11.20 ± 0.56) and solitarious locusts (12.18 ± 2.52; *t*_4.4_ = 0.38, *p* = 0.721). Therefore, normalizing the frequency response profiles to the maximum response did not introduce any major scaling distortions in the data between phases and we were able to compare the overall pattern of the electrophysiological response across all frequencies and sound levels. The baseline activity in the recordings was 8.58 ± 0.87% of the maximum response; any response above this level indicates a neural response to the presented stimulus.

We found that gregarious locusts showed a steeper decline in responsiveness for frequencies above approximately 10 kHz, whereas solitarious locusts retained a much stronger relative electrophysiological response at these higher frequencies (figure 4). For example, at 85 dB SPL, the electrophysiological response of gregarious locusts is below 60% of its maximum response (approx. 100% from 4 to 8 kHz) at frequencies greater than 13 kHz, whereas the response of solitarious locusts is above 60% at 85 dB SPL (figure 4*b*). At lower sound levels, e.g. 50 dB SPL, solitarious locusts have a larger electrophysiological response at nearly all frequencies (figure 4*b*).

To further understand the neural response, we analysed the RMS of neural activity in the auditory nerve with increasing sound intensity across representative frequencies (figure 5 and electronic supplementary material, table S1). Both phases reached greater than 95% of their maximum electrophysiological response between 4 and 8 kHz. However, for frequencies under 10 kHz, gregarious locusts reached a greater RMS response at lower sound levels. For example, at 3 kHz, the response of the gregarious locusts reached saturation at approximately 85 db SPL, whereas the response of solitarious locusts still increased with greater sound intensity up to the experimental maximum of 95 db SPL. Illustrating the point, at 80 dB SPL, there was a significant difference between phases, but at 90 dB the degree of difference between phases had diminished and they were no longer significantly different (3 kHz 80 dB, *F*_1,27_ = 6.519, *p* = 0.017; 90 dB, *F*_1,27_ = 3.511, *p* = 0.07; electronic supplementary material, table S1). At 10 kHz, there were no significant differences between the phases at any sound intensity (e.g. 80 dB SPL, *F*_1,27_ = 0.47, *p* = 0.5; electronic supplementary material, table S1; figure 5*c*). For high frequencies, such as 15 kHz, solitarious locusts had a significantly larger relative response across the majority of the sound intensity range reaching a maximum difference of 80 versus 65% of maximum (80 dB SPL, *F*_1,27_ = 10.37, *p* = 0.003; electronic supplementary material, table S1; figures 4 and 5*d*). There were no significant differences for frequency or sound intensity when comparing the data by sex rather than phase (electronic supplementary material, table S1).

### (d) Latency of response

**Figure 6.**
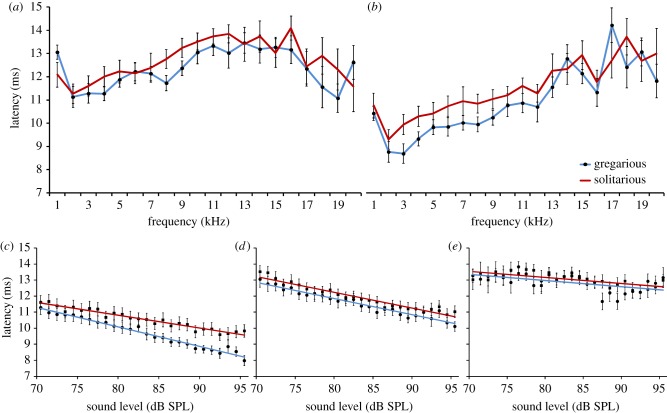
Latency to the initiation of the electrophysiological response at (*a*) 70 dB SPL and (*b*) 90 dB SPL and across 70–90 dB SPL at (*c*) 3 kHz, (*d*) 10 kHz, and (*e*) 15 kHz. The blue line with black dots is data from gregarious locusts, and the red line is solitarious locusts. (Online version in colour.)

There was a slight increase in latency to sound from 11 to 13 ms for sounds played at 70 dB SPL with increasing sound frequency up to 12 kHz, after which, the latency with higher frequencies remained close to 12 ms, for all locusts (figure 6*a*). At 90 dB SPL, there was a shorter latency to sound at lower frequencies which increased progressively as sound frequency increased (70 versus 90 dB: 3 kHz: *t* = 13.48, *p* < 0.001; 10 kHz: *t* = 10.02, *p* < 0.001; figure 6*a,b*). Gregarious locusts had shorter latencies than solitarious locusts for the lower frequencies (2–12 kHz), though this was only significantly different at 3 kHz (90 dB: 3 kHz, *F* = 5.9, *p* = 0.022; 4 kHz, *F* = 3.6, *p* = 0.069; 7 kHz, *F* = 2.4, *p* = 0.13; 10 kHz, *F* = 0.6 *p* = 0.47; figure 6*c,d*). For higher frequencies, there was no difference with phase (e.g. at 90 dB 15 kHz, *F* = 1.3 *p* = 0.27) and little decrease in latency with increasing sound levels (15 kHz: *t* = 10.02, *p* = 0.314; figure 6*e*).

## 4. Discussion

Locust hearing is sensitive over at least 1–30 kHz [22,27]. We found the greatest tympanal movement and electrophysiological response between 4 and 8 kHz. There were significant differences between phases for both these parameters (table 1).

**Table 1.** Comparison of hearing between solitarious and gregarious locusts.

	solitarious	gregarious
tympanal	7.5% wider: 1.56 ± 0.04 mm	width: 1.45 ± 0.02 mm
anatomy	→ affects the travelling wave	length: no significant difference
tympanal biomechanics	peak displacement 4–7 kHz	peak displacement 4–7 kHz
	*larger* membrane displacement, double for some frequencies (e.g. 0.31 ± 0.06 µm Pa^−1^ at 5 kHz)	*smaller* membrane displacement → needs 6 dB louder sound to move as much as solitarious
neurophysiology	peak response 4–8 kHz	peak response 4–8 kHz
	greater response at lower sound levels across frequencies	shorter latency, faster response, for lower frequencies
	requires *louder* sound levels to reach maximum response for low frequencies	requires *lower* sound levels to reach maximum response for low frequencies
	*greater* response at high frequency	*reduced* response at high frequency

When considering the shape of the tympana, which is the initial receiver of the sound, we found a significant difference between phases in the membrane width, with solitarious locusts having wider membranes (table 1). The width axis is the direction in which the travelling wave occurs (electronic supplementary material, videos S1–S3). As tympanal width varied with phase and the length did not, we believe the width of the membrane is conserved based on phase and contributes to the greater displacement of the travelling wave. While, solitarious locusts are larger than gregarious locusts, females are larger than males [5]; however, the lack of a significant effect of sex on tympanum width suggests that this difference is not a simple consequence of the relative body sizes of the two phases. The tympanal membrane was significantly longer in females than males, which we attribute to the larger size of the females, and it is not obviously reflected in their hearing ability. Furthermore, there were no significant sex-related differences in membrane movement or electrophysiological response. Ultimately, the displacement of the membrane from the travelling wave leads to the electrophysiological response of the auditory receptor nerve.

The overall neural response suggests that gregarious animals are highly preferentially tuned to frequencies below 10 kHz. Solitarious locusts, by contrast, exhibit a flatter response (figure 4). The greater movement of the membrane at the lower frequencies did not directly correlate with a larger neural response at these frequencies, a somewhat surprising result. By contrast, we saw shorter neural latency and a greater percentage response at lower sound levels for gregarious animals, not solitarious locusts. We suggest instead that larger movements at lower frequencies may correspond to more complex movements of Müller's organ, which in turn could enable better discrimination between different sound intensities. At lower frequencies, the frequency determination is more complicated owing to several types of neurons and attachment points along the membrane [24–25,31]. Müller's organ itself has differential movement with sound, with some of the receptor neurons terminating in the organ and not reaching the tympanal membrane [31]. Therefore, while there are also larger membrane deflections at lower frequencies, these deflections will differentially affect several types of sensory neurons at multiple attachment sites.

Conversely, at higher frequencies (greater than 12 kHz), frequency determination derives from one type of neuron attached at the PV [32] and so the link between movement and neural response can be more directly made. We therefore suggest that the greater displacement measured only affects the one neuron type at the PV, and so corresponds to the larger electrophysiological response. The exact mechanism of transduction is still not perfectly understood; though, it is suggested to be due to the travelling waves that occur across the tympanal membrane in response to sound leading to mechanical distortion and hence excitation of auditory afferents [27,28]. Our results measure the overall response of the auditory nerve, downstream of Müller's organ and not specific neurons, and so it is possible that individual neurons have different responses. Recordings made from individual auditory afferents would be required to establish how the response characteristics of the different classes of auditory afferents differ between phases.

The differing hearing ability of the two phases (table 1) appears to reflect the requirements of their lifestyles. Solitarious locusts, by definition, cannot rely on group protection from other locusts; they must rely on early detection and evasion to avoid predators. Solitarious locusts fly at night [10,11], and hence are potentially at much greater risk from predation by bats [22,33]. Our results suggest that solitarious locusts are also more responsive to the higher frequencies used by bats in their echolocation calls. Similarly, a visual neuron in locusts that detects objects on collision course, the descending contralateral movement detector (DCMD), shows a circadian variation in responsiveness that varies between phases, with the DCMD of solitarious locusts showing maximum responsiveness at and just after expected dusk [34], when they would be most at risk from nocturnal predators.

The sensory environment of gregarious locusts, however, is dominated by the presence of other locusts, compromising their ability to detect distant or weak stimuli and perhaps increasing the need to process fast changing information from close neighbours. As a consequence, gregarious locusts are better at processing and are less likely to habituate to looming objects (e.g. other flying locusts) and have larger brains, dedicated to higher processes. In addition, our results found gregarious locusts have faster response times to sounds (low frequency 1–8 kHz) and reach their maximum response with lower sound levels (table 1). Furthermore, gregarious locusts are more active during the day and so may need a stronger detection of birds. Responses to birds by other arthropods that are predated upon by birds, such as spiders, have been reported previously, for example, a singing bird indicates its presence in the environment [35,36] and butterflies hear the wing beats of birds [37]. Future work could also test whether locusts respond to the sound of bird wing beats and calls.

Ultimately, the locust's differing sensory abilities show an impressive epigenetic response for an animal to fit its sensory needs to the environment. For locusts, the adult morphology is a long-term, non-reversible, effect derived from environmental conditions prior to development and during early larval life. Behaviour and its underlying neural basis are more labile and can be altered in response to more immediate conditions. The auditory abilities of locusts depend on both the long-term development of body morphology shaping the structure of the tympanum and the properties of the auditory afferents innervating it. Therefore, the aspects of their hearing that do not change are also of interest as a conserved element that is not susceptible to phenotypic plasticity. Both morphologies retain peak sensitivity between about 4–8 kHz (table 1), which could be important for avoiding avian predators or for a historical function to hear mating calls.

Our results show a small morphological difference in the relative width of the ear membrane exists between solitarious and gregarious phase locusts and that may translate to a measurable effect on the membrane's vibratory properties and ultimately the neural responses. We have hypothesized how the resulting change in neural information presented may relate to how the different phases live in the environment. In the future, behavioural studies would aid the understanding of the two locust phases' hearing and establish how they use this epigenetic response in their interaction with the environment.

## Data accessibility

The data used in this paper is published on Dryad, doi:10.5061/dryad.4bm76.

## Supplementary Material

Table 1: Electrophysiological Relative Responses

## References

[1] Crispo E 2010 The evolution of phenotypic plasticity in response to anthropogenic disturbance. Evol. Ecol. Res. 12, 47–66.

[2] West-Eberhard MJ 1989 Phenotypic plasticity and the origins of diversity. Annu. Rev. Ecol. Syst. 20, 249–278. (10.1146/annurev.es.20.110189.001341)

[3] Dingemanse NJ Wolf M 2013 Between-individual differences in behavioural plasticity within populations: causes and consequences. Anim. Behav. 85, 1031–1039. (10.1016/j.anbehav.2012.12.032)

[4] Faulk C Dolinoy DC 2011 Timing is everything, the when and how of environmentally induced changes in the epigenome of animals. Epigenetics 6, 791–797. (10.4161/epi.6.7.16209) 21636976 PMC3230539

[5] Uvarov BP 1966 Grasshoppers and locusts: a handbook of general acridology. Cambridge, UK: published for the Anti-Locust Research Centre by Cambridge.

[6] Uvarov BP 1977 Grasshoppers and Locusts, vol. 2 London, UK: Centre for Overseas Pest Research.

[7] Pener MP Simpson SJ 2009 Locust phase polyphenism: an update. Adv. Insect Physiol. 36, 1–272. (10.1016/S0065-2806(08)36001-9)

[8] Simpson SJ McCaffery AR Hagele BF 1999 A behavioural analysis of phase change in the desert locust. Biol. Rev. Camb. Phil. Soc. 74, 461–480. (10.1017/S000632319900540X)

[9] Blackburn LM Ott SR Matheson T Burrows M Rogers SM 2010 Motor neurone responses during a postural reflex in solitarious and gregarious desert locusts. J. Insect Physiol. 56, 902–910. (10.1016/j.jinsphys.2010.04.011) 20416321

[10] Steedman A. (ed.) 1990 *Locust Handbook*, 3rd edn. Chatham, UK: Natural Resources Institute.

[11] Ely SO Njagi PGN Bashir MO El-Amin SET Hassanali A 2011 Diel behavioural activity patterns in adult solitarious desert locust, *Schistocerca gregaria*. Psyche 2011 (10.1155/2011/459315)

[12] Sword GA 1999 Density-dependent warning coloration. Nature 397, 217 (10.1038/16609)

[13] Ellis PE 1959 Learning and social aggregation in locust hoppers. Anim. Behav. 7, 91–106. (10.1016/0003-3472(59)90037-5)

[14] Roessingh P Simpson SJ 1994 The time-course of behavioral phase-change in nymphs of the desert locust, *Schistocerca gregaria*. Physiol. Entomol. 19, 191–197. (10.1111/j.1365-3032.1994.tb01042.x)

[15] Rogers SM Harston GWJ Kilburn-Toppin F Matheson T Burrows M Gabbiani F Krapp HG 2010 Spatiotemporal receptive field properties of a looming-sensitive neuron in solitarious and gregarious phases of the desert locust. J. Neurophysiol. 103, 779–792. (10.1152/jn.00855.2009) 19955292 PMC2822700

[16] Ochieng SA Hallberg E Hansson BS 1998 Fine structure and distribution of antennal sensilla of the desert locust, *Schistocerca gregaria* (Orthoptera : Acrididae). Cell Tissue Res. 291, 525–536. (10.1007/s004410051022) 9477309

[17] Rogers SM Matheson T Despland E Dodgson T Burrows M Simpson SJ 2003 Mechanosensory-induced behavioural gregarization in the desert locust *Schistocerca gregaria**.* J. Exp. Biol. 206, 3991–4002. (10.1242/jeb.00648) 14555739

[18] Ott SR Rogers SM 2010 Gregarious desert locusts have substantially larger brains with altered proportions compared with the solitarious phase. Proc. R. Soc. B 277, 3087–3096. (10.1098/rspb.2010.0694) PMC2982065 20507896

[19] Horridge GA 1961 Pitch discrimination in locusts. Proc. R. Soc. Lond. B 155, 218–231. (10.1098/rspb.1961.0067)

[20] Michelsen A 1971 Physiology of locust ear. 1. Frequency sensitivity of single cells in isolated ear. Z. Vergl. Physiol. 71, 49–62. (10.1007/BF01245154)

[21] Michelsen A 1971 Physiology of locust ear. 2. Frequency discrimination based upon resonances in tympanum. Z. Vergl. Physiol. 71, 63–101. (10.1007/BF01245155)

[22] Robert D 1989 The auditory-behavior of flying locusts*.* J. Exp. Biol. 147, 279–301.

[23] Haskell PT 1957 The influence of flight noise on behaviour in the desert locust *Schistocerca gregaria* (Forsk). J. Insect Physiol. 1 52–75. (10.1016/0022-1910(57)90023-9)

[24] Michelsen A Rohrseitz K 1995 Directional sound processing and interaural sound-transmission in a small and a large grasshopper. J. Exp. Biol. 198, 1817–1827. 9319725 10.1242/jeb.198.9.1817

[25] Gray EG 1960 The fine structure of the insect ear. Phil. Trans. R. Soc. Lond. B 243, 75–94. (10.1098/rstb.1960.0005)

[26] Michel K Petersen M 1982 Development of the tympanal organ in larvae of the migratory locust (*Locusta migratoria*). Cell Tissue Res. 222, 667–676. (10.1007/BF00213864) 7060108

[27] Windmill JFC Gopfert MC Robert D 2005 Tympanal travelling waves in migratory locusts. J. Exp. Biol. 208, 157–168. (10.1242/jeb.01332) 15601886

[28] Windmill JFC Bockenhauer S Robert D 2008 Time-resolved tympanal mechanics of the locust. J. R. Soc. Interface 5, 1435–1443. (10.1098/rsif.2008.0131) 18522928 PMC2607351

[29] Roessingh P Simpson SJ James S 1993 Analysis of phase-related changes in behavior of desert locust nymphs. Proc. R. Soc. Lond. B 252, 43–49. (10.1098/rspb.1993.0044)

[30] Schartau W Leidescher T 1983 Composition of the hemolymph of the tarantula *Eurypelma californicum*. J. Comp. Physiol. 152, 73–77. (10.1007/BF00689730)

[31] Stephen RO Bennet-Clark HC 1982 The anatomical and mechanical basis of stimulation and frequency-analysis in the locust ear. J. Exp. Biol. 99, 279–314.

[32] Jacobs K Otte B Lakes-Harlan R 1999 Tympanal receptor cells of *Schistocerca gregaria*: correlation of soma positions and dendrite attachment sites, central projections and physiologies. J. Exp. Zool. 283, 270–285. (10.1002/(SICI)1097-010X(19990215)283:3<270::AID-JEZ5>3.0.CO;2-C)

[33] Dawson JW Leung FH Robertson RM 2004 Acoustic startle/escape reactions in tethered flying locusts: motor patterns and wing kinematics underlying intentional steering. J. Comp. Physiol. A 190, 581–600. (10.1007/s00359-004-0521-8) 15127218

[34] Gaten E Huston SJ Dowse HB Matheson T 2012 Solitary and gregarious locusts differ in circadian rhythmicity of a visual output neuron. J. Biol. Rhythms 27, 196–205. (10.1177/0748730412440860) 22653888

[35] Lohrey AK Clark DL Gordon SD Uetz GW 2009 Antipredator responses of wolf spiders (Araneae: Lycosidae) to sensory cues representing an avian predator*.* Anim. Behav. 77, 813–821. (10.1016/j.anbehav.2008.12.025)

[36] Gordon SD Uetz GW 2012 Environmental interference: impact of acoustic noise on seismic communication and mating success. Behav. Ecol. 23, 707–714. (10.1093/beheco/ars016)

[37] Fournier JP Dawson JW Mikhail A Yack JE 2013 If a bird flies in the forest, does an insect hear it? Biol. Lett. 9, 20130319 (10.1098/rsbl.2013.0319) 23945205 PMC3971667

