# Supplementary material for: Listening to the environment: hearing differences from an epigenetic effect in solitarious and gregarious locusts
Source: Proc Biol Sci. 2014 Nov 22;281(1795):20141693. doi: 10.1098/rspb.2014.1693 (PMC4213621; doi:10.1098/rspb.2014.1693)
Supplement: Table 1: Electrophysiological Relative Responses [file rspb20141693supp1.pdf]

Table 1: Electrophysiological Relative Responses

Phase		Gregarious Average % (± S.E)	Solitarous Average % (± S.E)	F (1,27)	p
**Frequency**	**Decibel**				
3kHz	50	34.2 ± 2.0	35.1 ± 2.2	0.076	0.79
	60	48.4 ± 2.9	48.8 ± 3.4	0.007	0.94
	70	68.9 ± 2.4	63.4 ± 3.2	1.509	0.23
	80	83.1 ± 1.5	75.3 ± 2.2	6.519	0.017
	90	91.0 ± 1.6	86.2 ± 1.6	3.511	0.072
5kHz	50	28.6 ± 1.2	34.9 ± 3.0	2.663	0.12
	60	43.7 ± 1.6	46.7 ± 2.8	0.685	0.42
	70	63.8 ± 1.9	63.5 ± 2.8	0.005	0.35
	80	86.2 ± 1.6	83.0 ± 2.6	0.842	0.37
	90	99.9 ± 1.4	94.9 ± 1.9	3.376	.077*
8kHz	50	28.3 ± 1.7	31.7 ± 2.2	1.09	0.31
	60	45.2 ± 2.4	43.0 ± 2.5	0.313	0.58
	70	67.2 ± 2.5	61.7 ± 2.9	1.65	0.21
	80	90.0 ± 2.2	84.9 ± 2.3	2.085	0.16*
	90	100.2 ± 1.2	95.9 ± 1.4	8.922	0.006
10kHz	50	24 ± 2.3	25.3 ± 1.5	0.168	0.69
	60	34.9 ± 3.1	37.4 ± 1.7	0.38	0.54
	70	55.6 ± 4.2	57.1 ± 2.5	0.072	0.79
	80	76.4 ± 3.6	79.8 ± 2.6	0.467	0.50
	90	90.6 ± 2.1	95.1 ± 1.6	2.366	0.14
15kHz	50	19.6 ± 1.1	24.6 ± 1.9	3.833	0.061
	60	22.7 ± 1.3	28.0 ± 1.6	4.908	0.035
	70	32.5 ± 1.4	40.4 ± 1.9	8.711	0.006
	80	45.5 ± 2.2	57.7 ± 2.6	10.369	0.003
	90	64.3 ± 2.9	79.3 ± 2.1	11.109	0.003
18kHz	50	21.1 ± 1.2	21.9 ± 1.9	0.1	0.76
	60	22.5 ± 1.4	24.8 ± 2.0	0.738	0.40
	70	28.7 ± 1.4	34.4 ± 1.9	4.359	0.047
	80	44.6 ± 3.6	52.8 ± 2.6	2.593	0.12*
	90	61.2 ± 3.4	71.1 ± 2.3	2.985	0.10*

* indicates that nearby decibel levels were significantly different though this point was only close, they do not have overlapping S.E. bars.

Sex		Male Average % (± S.E)	Female Average % (± S.E)	F (1,27)	p
**Frequency**	**Decibel**				
3kHz	50	33.0 ± 2.1	37.1 ± 1.9	1.479	0.23
	60	47.7 ± 2.6	50.2 ± 3.7	0.311	0.58
	70	66.6 ± 2.5	65.3 ± 3.5	0.085	0.77
	80	79.7 ± 1.7	78.1 ± 2.6	0.232	0.63
	90	90.3 ± 1.7	85.5 ± 1.2	3.234	0.084
5kHz	50	30.3 ± 1.9	34.3 ± 3.1	1.019	0.32
	60	44.5 ± 2.4	46.2 ± 2.1	0.201	0.66
	70	63.3 ± 2.3	64.1 ± 2.6	0.046	0.83
	80	84.8 ± 2.3	84.2 ± 2.1	0.025	0.88
	90	98.8 ± 2.0	95.3 ± 1.3	1.45	0.24
8kHz	50	26.1 ± 1.4	36.1 ± 2.0	13.56	0.001
	60	41.0 ± 1.5	48.3 ± 3.2	3.9	0.059
	70	62.8 ± 2.2	66.6 ± 3.4	0.726	0.40
	80	88.3 ± 2.1	86.0 ± 2.6	0.395	0.54
	90	100.4 ± 1.5	96.9 ± 1.5	2.023	0.17
	50	22.6 ± 1.9	27.7 ± 1.6	2.686	0.11

10kHz	60	3.4 ± 2.3	39.3 ± 2.6	1.821	0.19
	70	52.9 ± 3.4	61.2 ± 3.0	2.376	0.14
	80	74.3 ± 3.1	83.6 ± 2.6	3.754	0.063
	90	92.8 ± 1.8	93.3 ± 2.1	0.022	0.88
15kHz	50	20.9 ± 1.5	24.0 ± 1.9	1.301	0.26
	60	24.0 ± 1.5	27.5 ± 1.7	1.932	0.18
	70	35.6 ± 1.9	38.0 ± 1.8	0.617	0.44
	80	51.7 ± 3.0	52 ± 2.3	0.006	0.94
	90	72.0 ± 3.6	74.0 ± 1.5	0.129	0.72
18kHz	50	20.6 ± 1.4	22.8 ± 1.9	0.718	0.40
	60	21.5 ± 1.3	26.8 ± 2.0	1.044	0.054
	70	29.5 ± 1.6	24.7 ± 1.7	3.495	0.073
	80	46.6 ± 3.0	52.2 ± 3.5	1.107	0.30
	90	65.2 ± 2.8	66.3 ± 3.7	0.028	0.87
